# Supplementary figures and images for: Genome-Wide Analysis of LncRNA in Bovine Mammary Epithelial Cell Injuries Induced by Escherichia Coli and Staphylococcus Aureus
Source: Int J Mol Sci. 2021 Sep 8;22(18):9719. doi: 10.3390/ijms22189719 (PMC8470725; doi:10.3390/ijms22189719)

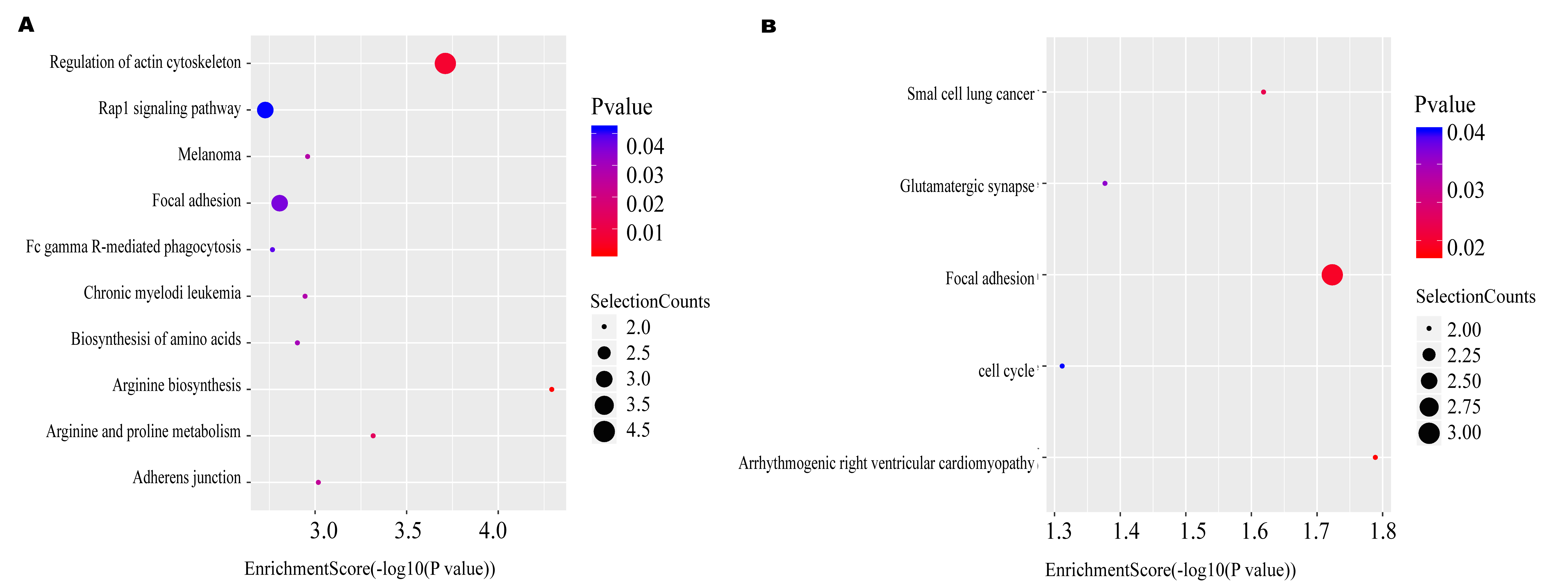

Supplement: Supplementary file 1 [file ijms-22-09719-s001.zip › Figure S2 The top 10 KEGG pathways enriched for the targets of significantly upregulated expressed lncRNAs..tif]
